# Mellitin peptide quantification in seasonally collected crude bee venom and its anticancer effects on myelogenous K562 human leukaemia cell line

**DOI:** 10.1186/s12906-023-03897-x

**Published:** 2023-04-25

**Authors:** Maher Obeidat, Ihab F. Al-khraisat, Da’san M. M. Jaradat, Bayan Y. Ghanim, Qasem M. Abdallah, Duaa Abu Arqoub, Duaa Sabbah, Ola M. Al-Sanabra, Tawfiq Arafat, Nidal A. Qinna

**Affiliations:** 1 Department of Medical Laboratory Analysis, Faculty of Science, Al-Balqa Applied University, Al-Salt, Jordan; 2 Department of Chemistry, Faculty of Science, Al-Balqa Applied University, Al-Salt, Jordan; 3 Department of Pharmacology and Biomedical Sciences, University of Petra Pharmaceutical Center (UPPC), Faculty of Pharmacy and Medical Sciences, University of Petra, P.O. Box 961343, Amman, Jordan; 4 Jordan Center for Pharmaceutical Research (JCPR), Amman, Jordan

**Keywords:** Bee venom, Apitherapy, Cytotoxicity, Apoptosis, Cell arrest, Melittin

## Abstract

**Background:**

Apitherapy is an emerging field in cancer research, particularly in developing communities. The potency of Melittin (MEL), a major constituent in bee venom is accounted for the cytotoxic capacity against cancer cells. It is postulated that the genotype of bees and the time of venom collection influences its specific activity against certain types of cancer.

**Method:**

Hereby, Jordanian crude bee venom (JCBV) was collected during different seasons of the year, specifically spring, summer and autumn and investigated for in vitro antitumour effects. Venom collected during springtime comprised the highest quantity of MEL in comparison to venom collected some other time. Springtime-collected JCBV extract and MEL were tested on an immortal myelogenous leukaemia cell line, namely K562 leukemic cells. Treated cells were examined for cell modality via flow cytometry analysis and cell death mediating gene expressions.

**Results:**

Springtime-collected JCBV extract and MEL showed an IC_50_ of 3.7 ± 0.37 μg/ml and 1.84 ± 0.75 μg/ml, respectively. In comparison to JCBV and positive control, MEL-treated cells exhibited late apoptotic death with a moderate cellular arrest at G0/G1 and an increase of cell number at G2/M phase. Expression of NF-κB/MAPK14 axis was inhibited in MEL and JCBV-treated cells, as well as expression of c-MYC and CDK4. Moreover, marked upregulation in ABL1, JUN and TNF was observed. In conclusion, springtime-collected JCBV showed the highest content of MEL while both JCBV and pure MEL showed apoptotic, necrotic, and cell cycle arrest efficiency against K562 leukemic cells.

**Conclusion:**

Integration of bee venom in chemotherapy needs more investigation and should be carefully translated into clinical use. During such translation, the correlation of bee genotype, collection time and concentration of MEL in CBV should be profiled.

## Background

Bee venom (BV) and honey that are considered ancient means of healing and therapy for many pathologies and symptoms, nevertheless, have been used in several products and drugs for their various indications. A lot of evidences indicate that humans have historically used BV in the treatment of many infections accounted to its antimicrobial peptides. Bee Venom is produced by bees when feeling threatened and is often created in defense against predators. It is poisonous to both animals and humans and shows an allergic effect. Among the popular applications of bee stings is joint and bone pain, acting as an anti-inflammatory intervention by placing the bees on the areas where the patient suffers [1]. Previous studies reported marked effects of BV on bacteria, fungus, viruses and cancers [2–4].

A large percentage of BV composition is Melittin (MEL), which is considered the most important water-soluble peptide of BV and exhibits antibacterial and cytolytic activities on cell membranes. Furthermore, BV containing MEL was found to inactivate the NF-κB pathway, induce apoptosis and inhibit metastasis in the prostate, colon, and mammary cancer cells [5]. It has also been reported that MEL causes cell lysis in leukaemia cells by pore formation, enhanced activity of death receptors, inhibition of signal transducers and activators of transcription (STAT) and BCL-2 pathways, and regulation of caspases [5].

Leukaemia is a group of cancerous diseases that affect the blood and tissues that produce blood. Due to abnormal proliferation and accumulation of leukemic cells, leukaemia reduces normal hematopoiesis. According to the Cancer Statistics Center, leukaemia is estimated to be accounted for 23.7 thousand deaths amongst the American Society in 2023, with around 60 thousand new cases. The incidence rates of leukemia rise annually to reach 14.1 in 2023 [6]. for 2.8 percent of new cases worldwide and accounts for 3.8 percent of total deaths in 2018, it is estimated that about 440 thousand new cases of leukaemia were diagnosed and about 310 thousand cancer deaths were caused by this disease worldwide [6]. Chronic Myeloid Leukaemia (CML) is a clonal myeloproliferative disorder of hematopoietic stem cells that accounts for about 20% of all adult leukaemia cases. CML is associated with large proliferation within the bone marrow and peripheral blood of white cell abnormalities which are categorized as major forms of acute and chronic leukaemia. Thus, there is an urgent need to search for natural resources to discover new therapeutic agents that may have a good potential in the selective killing of leukaemia cells.

Therefore, the current study investigates the chemical indifferences between Jordanian crude bee venom (JCBV) collected seasonally during the year. Furthermore, assesses the cytotoxic activity of bee venom against K562 CML leukaemia cells and determines the mode of cell death modality and underlying key genetic mediators.

## Methods

*Apis mellifera syriaca*, is the native honeybee subspecies of Jordan and much of the Levant. Jordanian Crude Bee venom (JCBV) was collected from native productive bee apiaries from areas located in Al-Anabtawi apiaries (32.14841767400204: 35.84738764283f295) which are located in the middle of Jordan about 15 km from the Jordanian capital Amman. The apiaries are also neighboring the city of Al-Salt and the city of Jerash, and are allocated to a mountainous region characterized by cold weather in winter, moderate in spring and autumn, and moderate to high in the middle of the summer season. A beehive containing about 8,000–10,000 bees was selected, and the collection process took place during daytime between 10 to 11 am so that the local temperature was not high, ranging between 20–25˚C in spring (April), 25–30˚C in summer (June) and 15–18˚C in fall season (October–November). The bee hives were left to recover for a week and collection was repeated again for four consecutive times per season. An in-house device was designed to mimic the principle of a bee stinging without harming and killing them. The 3 mm wires of the BV collector are connected to a battery of 12–15 V, 2 Amp; AC 25 V; 1200 Hz, to enable the generation of electrical impulses with frequency ranging from 50 to 1000 Hz. After each collection, the glass plate where yellowish gum-like venom was collected, was left to air-dry up into a white-yellowish crystal form. The formed crystals were then crushed into containers and preserved at -20 °C in dark and dry conditions to avoid autolysis (protease present in BV) until further analysis [7]. The yield of crystals varied between half a gram to a gram depending on the interval between collection frequencies.

### LC–MS-MS

In order to assure consistent drying of all collection, JCBV samples were lyophilized prior to further analysis using a lyophilizer (Heto dry winner, Thermo Fisher/USA) to do all measurements on dry basis. Venom crystals were dissolved in water, and then, LC–MS-MS studies were carried out using AB sciex 3200 MS detector; 25 °C oven temperature, chromatographic Column: ACE C8 (50 mm X 2.1) 5 μm; 25 °C separation temperature; and 1–2 ml/min flow rate for the mobile phase. For eluent solvent A; water, 0.5% mM NH_4_Cl with 0.1% Formic Acid, and 0.1% acetonitrile, as for eluent solvent B; air, water, and acetonitrile were used. Daughter fragment 712 was used and the gradient mixing solvent was carried out using solvents A and B.

### Cell culture and cytotoxicity assay

To determine the cytotoxic concentration of the MEL and JCBV, the compounds were tested in vitro on human myelogenous leukaemia K-562 cells (CCL-243, ATCC, USA) with 5-fluorouracil (5-FU) as a positive control. The selection of 5FU as a positive control was deemed crucial to ensure the validity of the experiment and to verify the reliability of the cytotoxicity assay, not to establish a comparison in terms of potency or mechanism of action with MEL or JCBV. A standard 5 mg MEL was purchased from Genscript with the following sequence GIGAVLKVLTTGLPALISWIK-RKRQQ-NH_2_ and purity of > 95% (HPLC Purity). K562 cell line was seeded (10^3^ cells/well) and cultured in RPMI-1640 medium (Caisson, USA) supplied with heat-inactivated FBS (10%), sodium pyruvate (1 mM), and L-glutamine (2 mM), in a humidified incubator at 37 °C enriched with 5% CO_2_. Serial dilution concentrations of test compounds (JCBV, MEL, 5-FU) were added and incubated for a time course of 96 h. Then, cell viability was tested using sulforhodamine B (SRB) assay following a protocol described previously with minor modifications [8]. Briefly, K562 cells were fixed at 4 ºC for 1 h with ice-cold Trichloroacetic acid solution (TCA, final concentration 10% w/v). Then the supernatant containing TCA was discarded and cells were washed 5 times with running tap water to ensure the removal of TCA residues before leaving to air-dry. The SRB solution (0.04% w/v in 1% acetic acid) was then added for 30 min before excess SRB dye is washed with 1% acetic acid. SRB dye was extracted from stained cells by Tris solution (10 mM, 100 µl/well), and absorbance was read using a multiwell plate spectrophotometer at a wavelength of 570 nm. The average absorbance of untreated K562 cells was taken as 100% survival. The experiment was done in triplicate and the IC_50_ values were calculated from each experiment [9].

### Flow cytometry

To determine the mode of death and effects on cell cycle induced by JCBV compared to MEL, K562 cells treated with JCBV and MEL where the cell death mechanism was determined by Annexin/PI assessment using flow cytometry. Cells were seeded at a concentration of 4 × 105 cells/T75 flask and exposed to an IC70 equipotent dose of JCBV and MEL (7.4 µg/ml and 3.6 µg/ml respectively). The selection of an IC70 dose was based on the premise that this dose would elicit discernible biological responses that are relevant to the mechanism of action. Lower doses may not generate substantial responses, leading to inaccurate measurements. Conversely, higher concentrations would result in the extermination of a significant proportion of cells, thereby limiting the availability of a sufficient number of cells for conducting apoptosis, cell cycle, and gene expression assessments. Utilizing distinct cell populations for each test would compromise the inter-test comparability of results. Doxorubicin (DOX 0.3 µg/ml) was used as a positive control, Doxorubicin was an essential control due to its pivotal role in both verifying the validity of the assay and in facilitating a comparison of apoptosis and cell-cycle responses between the treatments, thereby aiding in the investigation of the underlying mechanisms involved in the induction of cell death by MEL and JCBV. Moreover, based on our previous in-house research and data analysis, we concluded that the 0.3 µg/ml dose of doxorubicin, in similar assay conditions, is capable of inducing an approximately 70% of cell growth inhibition on the K562 cell line. Consequently, we chose this dose of doxorubicin as a positive control. After 96 h, cells were collected and prepared for Annexin/PI and Cell Cycle assessment according to the manufacturer’s instructions (Thermo Fischer, USA). Samples were analyzed using flow cytometry (BD FACS Canto II. USA).

### RNA extraction and RTqPCR

A pool of cells per group was obtained and homogenized by syringe and vortexing. Isolated pool samples of cell culture group homogenates were extracted for RNA using the RNeasy Mini Kit (QIAGEN, Hilden, Germany) according to the manufacturer's instructions. Extracted RNA samples were further quantified using a UV–Vis spectrophotometer equipped with a NanoDrop 2000c (Thermo Scientific, USA) to determine the integrity and purity of the RNA samples in terms of protein, and salt contaminants. RNA was test for quality in an agarose gel 1% (w/v) which was prepared by suspending agarose (Cleaver Scientific Ltd, UK) in 1X TBE buffer (Bio Basic, Canada) and adding 0.02 g/ml Ethidium bromide (Bio Basic, Canada). The gel was fitted in a horizontal gel electrophoresis tank (Cleaver Scientific Ltd, UK) and run at a voltage set to 120 V for 30 min. Thereafter, template RNA concentrations were set to 70 ng/μl and cDNA was reverse transcribed from RNA using Quantitech Reverse Transcriptase kit (QIAGEN, Germany) and Bioer GeneQ thermal cycler (Hangzhou, China). Transcribed cDNA was amplified and quantified by RTqPCR using SYBR Green qPCR reagent (New England, UK), using parameters described elsewhere [10]. The relative expressions of genes were assessed in comparison to housekeeping gene β-actin [11]. Table 1 shows primers designed via NCBI/Primer-Blast.

**Table 1:** Primers designed in the current study for amplification of cDNA of leukemic cells K562

Gene	Gene description	Primers (5’ → 3’)
*β-actin*	beta-actin (housekeeping gene)	F: ACCAACTGGGACGACATGGAG R: GTGAGGATCTTCATGAGGTAGTC
*MYC*	c-myc proto-oncogene product, recruits histone acetyltransferases	F: CTCTCAACGACAGCAGCCCG R: CCAGTCTCAGACCTAGTGGA
*RELA*	RELA proto-oncogene (p65), NF-kB subunit	F: AGGCTATCAGTCAGCGCATC R: TCCCCACGCTGCTCTTCTAT
*JUN*	JUN proto-oncogene, AP-1 transcription factor subunit	F: GAGCTGGAGCGCCTGATAAT R: CCCTCCTGCTCATCTGTCAC
*FOS*	forms a heterodimer with JUN	F: GCTGGCGTTGTGAAGACCAT R: TTGGTCTGTCTCCGCTTGGA
*BAX*	BCL-2 associated X-protein	F: TCAGGATGCGTCCACCAAGAAG R: TGTGTCCACGGCGGCAATCATC
*CAS9*	caspase 9 (initiator caspase)	F: CGAACTAACAGGCAAGCAGC R: GTCTTTCTGCTCGACATCACC
*CDK4*	Cyclin-dependent kinase 14	F: GTGTATGGGGCCGTAGGAAC R: CCATAGGCACCGACACCAAAT
*MAPK14*	member of the p38 mitogen-activated protein kinase (MAPK) family	F: TAACAGGATGCCAAGCCATGAG R: GCTTGGGCCGCTGTAATTC
*BCL-2*	B-cell lymphoma 2	F: ATACCATGATAGCGCCCTTG R: AATCACAGCGAACCTCTGCT
*TNFα*	tumor necrosis factor-alpha	F: CTCTTCTGCCTGCTGCACTTTG R: ATGGGCTACAGGCTTGTCACTC
*ABL1*	Philadelphia translocation	F: GACATGCCATAGGTAGCAATTTCCC R: ACATCACGCCAGTCAACAGTCTGG

The PCR reactions were carried out and fold-change amplification data were analyzed using the Rotor-GeneQ Pure Detection Series Software (Version 1., Build 94; 5-Plex) (QIAGEN, Germany). The fold regulation was considered significant when found over two-folds [12–14].

### Statistical analysis

Values are expressed as mean ± standard error of mean (SEM). Values were compared and analyzed using One-way ANOVA followed by post-hoc Tukey’s HSD test using Prism GraphPad 8.0.1. (California, USA). Results were considered significant for p-values ≤ 0.05.

## Results

### Chromatograph profiling

**Fig. 1:**
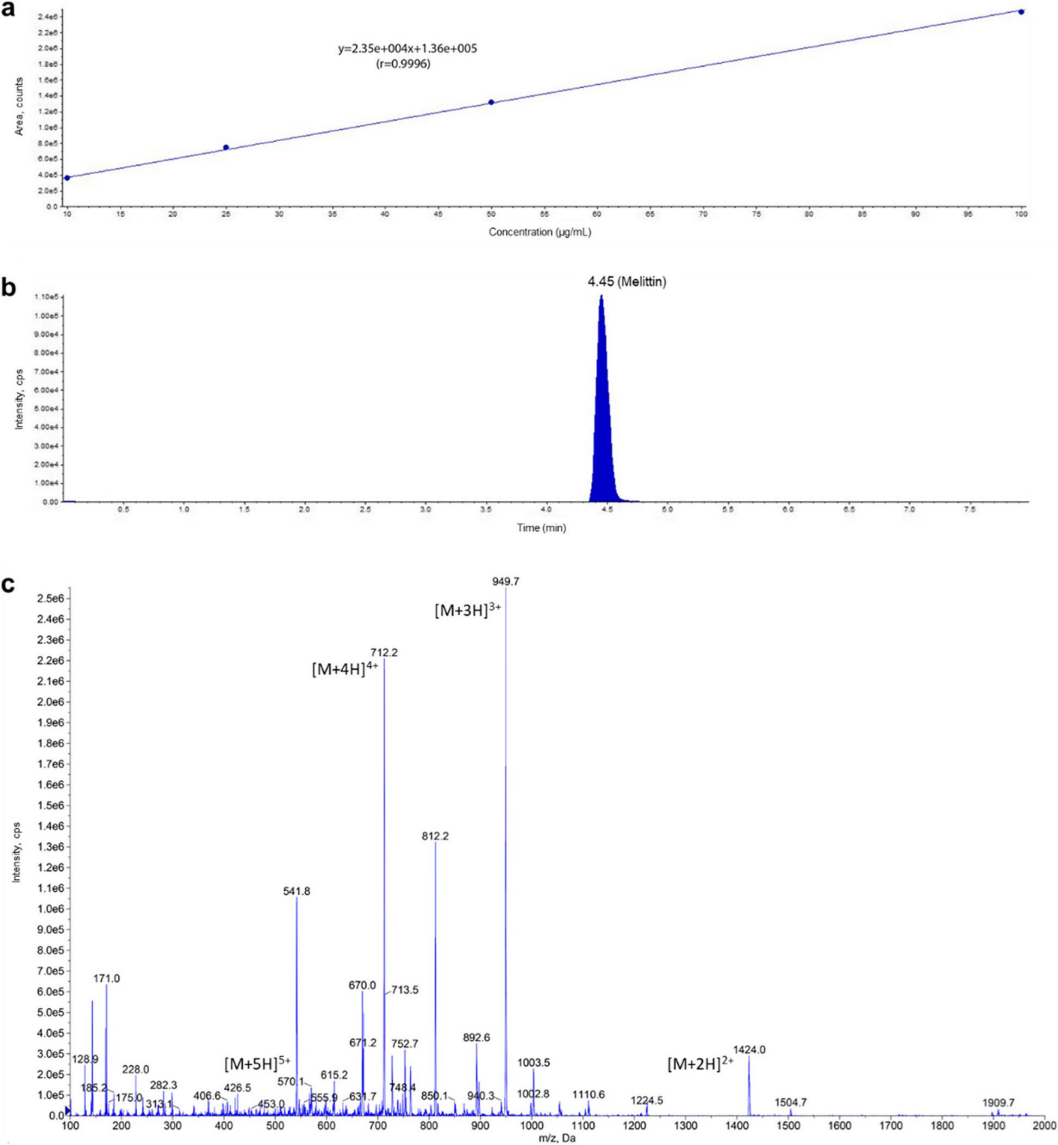
MS spectroscopy of Jordanian crude bee venom (JCBV) collected in Spring and Melittin (MEL) analytical standard. **a** Calibration curve of MEL using range of concentrations 10–100 ppm (R = 0.9999); **b** MEL spike found in springtime JCBV (RT = 4.5); **c** MS spectroscopy of JCBV collected in spring (1424 [M + 2H]2 + , 949.7 [M + 2H]3 + , 712.2 [M + 2H]4 + , and 570.1 [M + 2H]5 +)

To determine the efficacy and selectivity, MEL and BV collected using an electrical venom collector from Jordan in different seasons were investigated using the LC–MS-MS to determine the concentration of MEL in seasonally collected BV, the method validity was tested in terms of linearity using a calibration curve of MEL standard (Fig. 1a). Nevertheless, BV collected in springtime comprised the highest concentration of MEL which presented 25% of its constituents. The selected reaction monitoring (SRM) transition was picked from the main production at m/z 712 [M + 4H]^4+^ caused by the protonation of the side chains of four basic amino acid residues of MEL with retention time (tR) of 4.45 min (Table 2 and Fig. 1b). Table 1 shows the most abundant ion peaks of Melittin present in Jordanian crude bee venom during different seasons of the year. Nevertheless, according to the LCMS/MS analysis of seasonal BV, it was found that the concentration of MEL in JCBV collected in spring (Fig. 1c), represented one fourth of the constituents.

**Table 2:** The most abundant fragments of Melittin present in Jordan crude bee venom during different seasons

JCBV sample season	Ion	Formula	Result (Da)
Spring, Summer, Autumn	**[M + 2H]**^**2+**^	(MW + 2)/2	1424
Spring, Summer, Autumn	**[M + 3H]**^**3+**^	(MW + 3)/3	949.7
Spring, Summer, Autumn	**[M + 4H]**^**4+**^	(MW + 4)/4	712.2
Spring, Summer	**[M + 5H]**^**5+**^	(MW + 5)/5	570.1

### In-vitro cytotoxicity and cellular modality

**Fig. 2:**
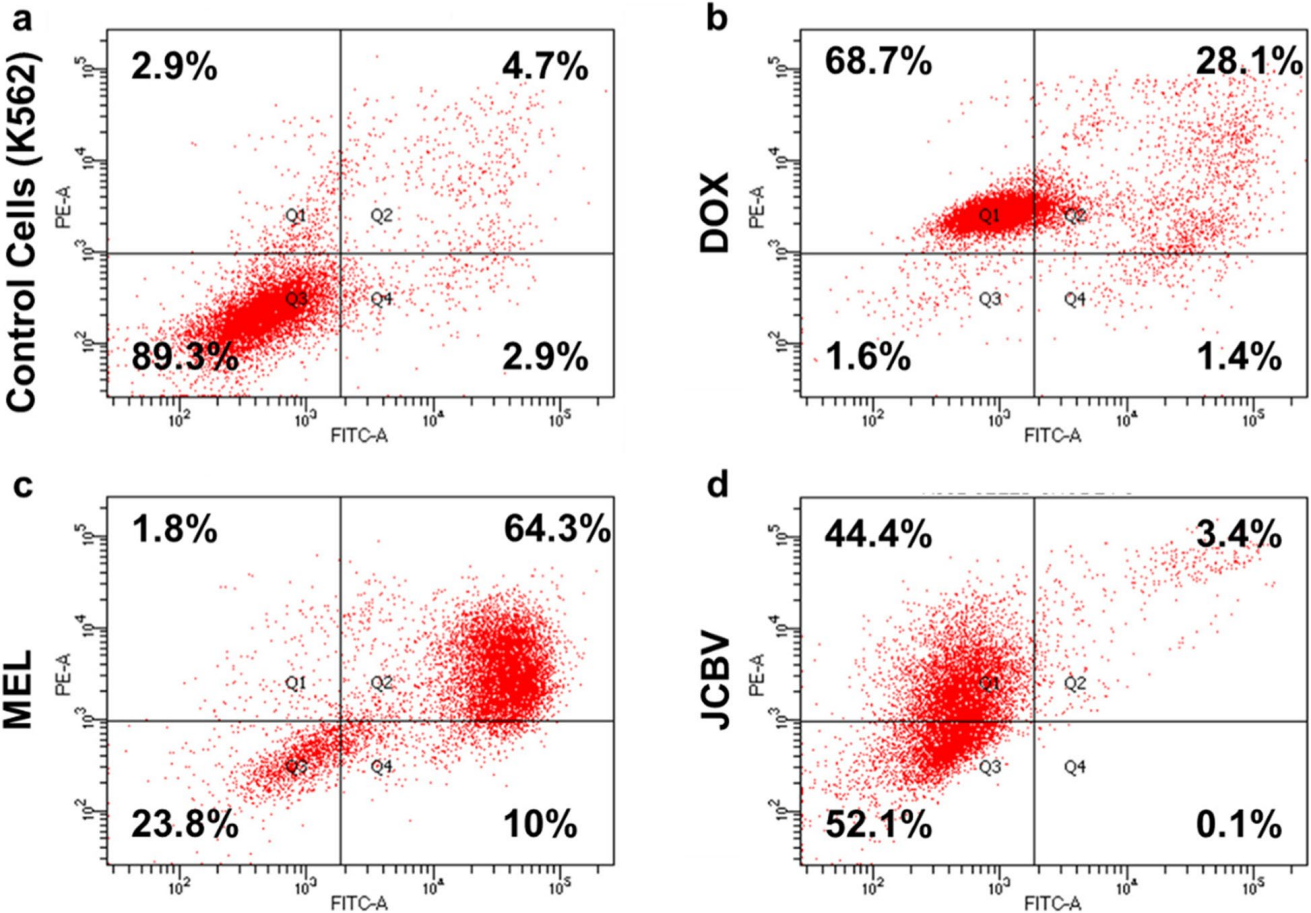
Flow cytometry dot plot of cellular modality in K562 cells. Dot plots of (**a**) untreated leukaemia cancer cells K562 (Control Cells); treated with (**b**) Doxorubicin 0.3 μg/ml (DOX); (**c**) Melittin 3.6 μg/ml (MEL); or (**d**) Jordanian crude bee venom 7.4 μg/ml (JCBV) stained with FITC-conjugated Annexin V and PI-stain. Plot quadrants indicate Q1: Necrosis, Q2: late apoptosis, Q3: healthy cells, Q4: early apoptosis. The analysis presented as the mean percentage of total cells of triplicate samples (*n* = 3)

**Fig. 3:**
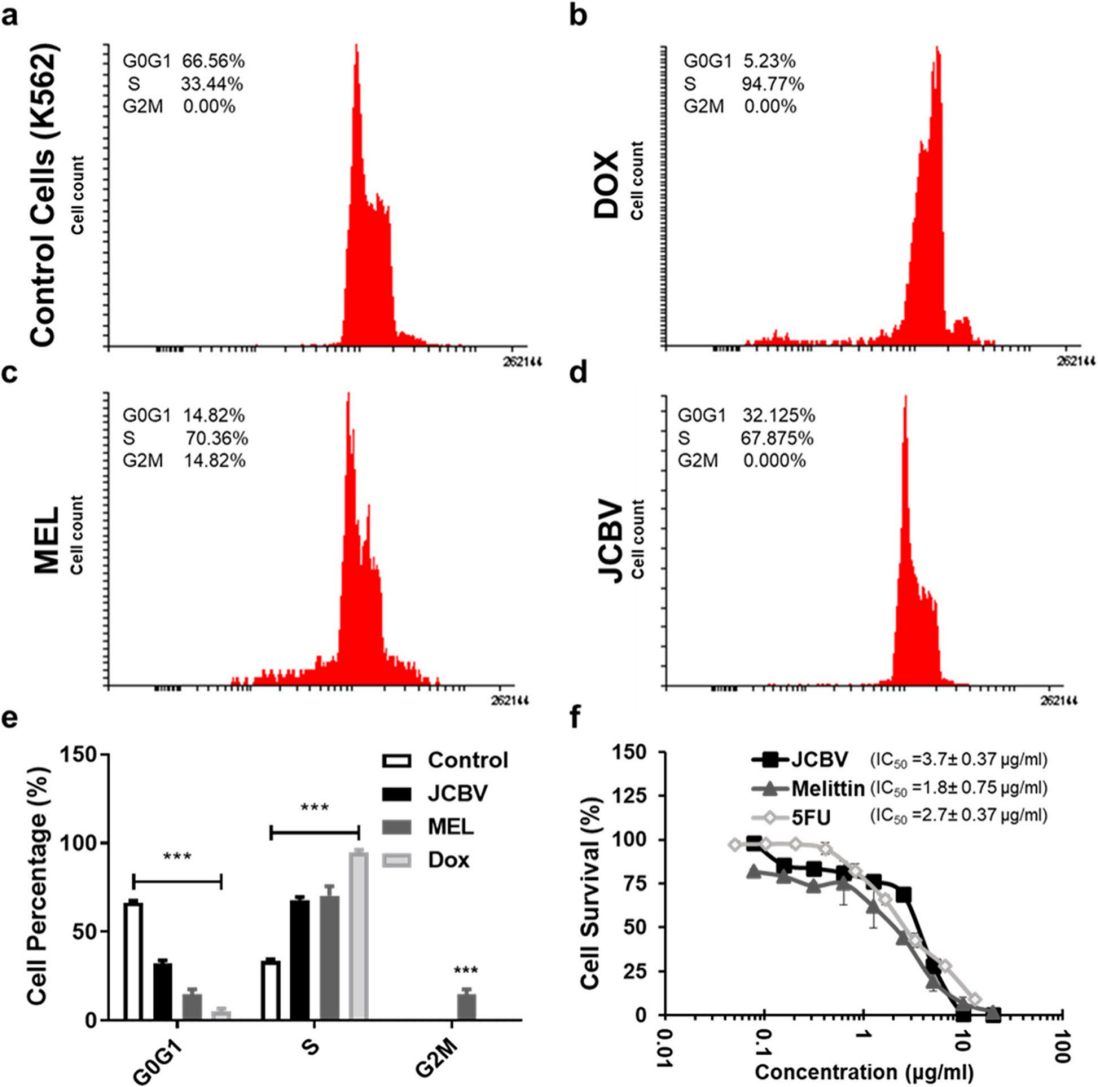
Cell cycle analysis. Flow cytometry cell cycle analysis of (**a**) untreated leukaemia cancer cells K562 (Control Cells); treated with (**b**) Doxorubicin 0.3 μg/ml (DOX); (**c**) Melittin 3.6 μg/ml (MEL); or (**d**) Jordanian crude bee venom 7.4 μg/ml (JCBV) (JCBV) stained with Propidium Iodide (PI). (**e**) Statistical analysis of cell cycle data is treated cells in comparison to control. (**f**) IC_50_ values of 5-FU, MEL, and JCBV on K562 leukaemia cell lines. Data are represented as the mean ± standard deviation (SD), and the means are of three independent replicates (*n* = 12). ***p-value ≤ 0.001 in comparison to control

Cytotoxicity assay results (Fig. 3f) showed that both JCBV and MEL were able to inhibit the growth of K562 cells by 50% at relatively low concentrations (3.7 ± 0.37 and 1.84 ± 0.75 μg/ml, respectively). The cellular death modality of leukaemia cell lines (K562) was studied upon exposure to MEL, JCBV and DOX. Figure 2a reveals that control untreated cell lines demonstrated a high percentage of healthy cells (89.3%). Treatment of K562 cells with a well-known chemotherapeutic agent, namely DOX (0.3 μg/ml) resulted in necrosis of cells as clustered in the first quadrant (average ~ 68.7%) (Fig. 2b) with mild clustering at the second quadrant which indicates late apoptosis of the cells (average ~ 28.1%). Treatment with MEL (IC70; 3.6 μg/ml) indicated that cells undergo late apoptosis (average ~ 64.3%) (Fig. 2c) unlike when treated with JCBV (IC70; 7.4 μg/ml), the cellular death modality was necrotic cell death to almost half of the population (average ~ 44.4%) (Fig. 2d).

The effect of treating K562 cells with MEL and JCBV on the cell cycle progression was evaluated by flow cytometry. K562 cells were exposed to our treatment; MEL, JCBV for 96 h. Cells treated with DOX were used as a positive control. Results indicated that MEL, JCBV and DOX showed a significant change in the progression of cells in cell cycle phases, compared to the untreated control group (Fig. 3a-e). As the percentage of treated cells was decreased in G0/G1 in a significant manner compared to the control untreated group (p < 0.001). Moreover, the cell percentage was increased significantly in the S phase in all treated groups compared to the control untreated group (*p* < 0.001). Interestingly, K562 cells treated with MEL showed a significant increase in the cell percentage in the G2/M phase, indicating G2/M arrest of MEL-treated cells (Fig. 3). Similar results were not evident in other cells treated with JCBV. Additionally, MEL treated cells showed a sub G0/G1 population of cells, indicating the apoptotic cell death of treated cells.

### Gene expression

**Fig. 4:**
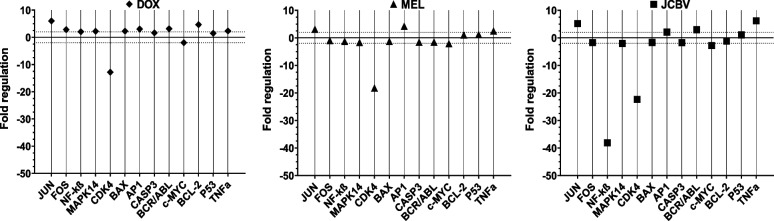
Expression of oncotic and cell survival encoding genes presented as relative fold change in K562 leukaemia cancer cell line treated with Doxorubicin 0.3 μg/ml (DOX) (red); Melittin 3.6 μg/ml (MEL) (blue) or Jordanian crude bee venom 7.4 μg/ml (JCBV) (green). Dotted horizontal lines resemble twofold regulation which was considered significant

The expression of the cell cycle regulator, namely CDK4 was markedly downregulated in all treated K562 cells despite the treatment (Fig. 4 and Table 3). Likewise, upregulation was observed in TNF and JUN (AP1) in all cell treatments. The expressions of the c-MYC gene were altered after MEL and JCBV treatment of K562 cells; c-MYC was downregulated. Treatment of K562 cells with DOX has exclusively induced the expression of BAX, BCL2, FOS, and NF-κB RELA (p65) genes (Fig. 4 and Table 3). Unlike MEL, treatment with JCBV induced the expression of ABL1 (BCR/ABL) gene and significantly downregulated the expression of NF-κB RELA and MAPK14 genes. The former genes were upregulated by DOX treatment.

**Table 3:** Fold regulation in genes expressed in several pathways after treatment of K562 leukaemia cells with Doxorubicin, Melittin, or Jordanian crude bee venom

Gene	Description	Fold Regulation*		
		**DOX**	**MEL**	**JCBV**
**Pro-apoptotic genes**				
***BAX***	BCL-2 Associated X-protein	2.26*	-1.32	-1.67
***CASP9***	Caspase 9 (initiator caspase)	1.65	-1.62	-1.77
**Cell signalling cytokine gene**				
***TNF***	Tumour necrosis factor	2.32*	2.44*	6.18*
**Anti-apoptotic gene**				
***BCL-2***	B-cell lymphoma 2	4.66*	1.02	-1.16
**Proto-oncogenes**				
***c- MYC***	Recruits histone acetyltransferases	-1.97	-2.14*	-2.80*
***NF-κB RELA***	Nuclear factor-kappa-B p65 subunit	2.06*	-1.33	-38.14*
***JUN (AP1)***	Forms the AP-1 early response transcription factor	6.01*	3.07*	5.15*
***FOS***	Forms a heterodimer with c- JUN	2.83*	-1.06	-1.72
**Oncogene**				
***ABL1 (BCR/ABL)***	ABL proto-oncogene 1, non-receptor tyrosine kinase	3.17*	-1.59	2.97*
**Cell cycle regulator**				
***CDK4***	Cyclin-dependent kinase 4	-12.81*	-18.26*	-22.38*
**Mitogen-activated protein kinases**				
***MAPK14 (p38)***	Member of the p38 MAPK family	2.23*	-1.72	-2.03*

^*^-2 ≥ fold-regulations ≥ 2 were considered significant [15]. Fold-regulations ≥ 2 were considered significant upregulation, and Fold-regulations ≤ -2 were considered significant downregulation

## Discussion

Apitherapy is a globally emerging field in cancer research, particularly in developing communities. Studies on the molecular mechanisms underlying the impact of honeybee venom and Melittin against cancer, particularly lymphoma, are still warranted. The active component of bee venom, namely Melittin comprises up to half of the bee venom dry weight [16]. Melittin has exhibited antitumor effects on several types of cancer including melanoma [17], glioblastoma [18], ovarian and breast cancers as well as others [19, 20].

The potency of Melittin regarding genotypes of bees and its specific activity against certain cancer types is also still under investigation. The current research highlights that the reduced concentration of MEL in JCBV might be linked to the time of collection or a high percentage of protease enzyme. Therefore, the correlation between collection time and concentration of MEL in JCBV should be investigated and might be attributed to the percentage of protease which may degrade and reduce MEL concentration [7].

In the current study, Melittin was examined for its potential cytotoxic effects against the cancerous K562 cells. The K562 cells are human CML cancer cell line that carries Philadelphia chromosome and can differentiate into resistant erythroleukemic cells. In line with recent reports which indicated that the exposure to different concentrations of MEL was considered cytotoxic to cells [21], the present study found that a concentration of 1.85 ± 0.75 µg/ml was capable of inducing growth inhibition to half of the population.

In the current study, after running chemical analysis, JCBV from spring season bared considerable levels of Melittin (Fig. 1), thus it was the candidate to be used in cytotoxicity assays. In vitro cytotoxicity found that the IC_50_ of JCBV was equivalent to two times the IC_50_ of MEL. Nevertheless, according to the LCMS/MS analysis of seasonal BV, it was found that the concentration of MEL in JCBV collected in spring, represented one fourth of the constituents. In the present study, MEL was found effective to inhibit the growth of leukemic cells by inducing apoptosis in cultured cells. Apoptosis is caused through a complex cascade of cellular executioners and is often regulated by internal and external cellular pathways [22]. MEL-induced apoptotic mode of cell death that was further confirmed with flow cytometry analysis which showed induction of apoptosis, unlike the crude bee venom extract. This could be explained by the breakage or damage in the DNA structure of treated cells, which prevent DNA replication. However, almost half of the cell population treated with JCBV expressed necrotic cell death, such event might be accounted to a lethal enzyme found in crude bee venom, namely phospholipase A2 (PLA2). PLA2 is a main constituent of bee venom which is activated by Melittin [23], and known to induce membrane phospholipid damage [24].

NF-κB is considered a negative apoptosis regulator that inhibits cell death signaling pathways, helps tumors to avoid cell death, builds drug resistance, and is a regulator of innate immunity. Despite that the anticancer activity of BV has been accounted to MEL through its induction of death receptor 3 and inactivation of NF-κB [4], the expression of NF-κB RELA (p65) was not altered in cells treated with MEL, however, treatment with JCBV suppressed its expression. The transcriptional factor NF-κB is extensively recognized as a vital biomarker in cancer pathogenesis due to its role in boosting cell renewal, and inducing chemotherapeutic resistance [25]. The relationship between NF-κB and BCR/ABL (ABL1) is long-lasting in Philadelphia positive leukaemia. For a long time, NF-κB was described as a crucial modulator in BCR/ABL signaling [25]. Expression of BCR/ABL was found upregulated in JCBV-treated cells, unlike MEL-treated cells where it was found unchanged. Such observation is prone to multiple speculations, mainly, that BCR/ABL is necrosis-mediated and necrotic cell death was noted in JCBV-treated cells not in MEL-treated [26]. Another explanation could be through c-MYC modulation of BCR-ABL expression, BCR/ABL is considered under the transcriptional control of the MYC oncogene [27], and c-MYC was found downregulated in the studied cellular model. The BCR/ABL fusion gene and its downstream signaling pathways including PI3K/AKT and Ras/Raf/MAPK pathways play important roles in malignant transformation. The expression and activity of MAPK pathway have been reported to be directly mediated by the BCR/ABL pathway signaling. MAPK is well-known for its antiapoptotic function, therefore, leading to an apoptosis-resistant phenotype [28]. Downregulation of MAPK14 in K562 cells was evident in JCBV-treated cells, thus inhibiting cell proliferation by subsequent downregulation of several oncogenes like c-MYC [29]. MEL, the peptide component of BV, has been re-ported to suppress the growth and migration of melanoma cells via MEL-induced inhibition of the PI3K/AKT/mTOR pathway and MAPK pathways [2, 3]. In the current study, MEL and JCBV resulted in the downregulation of c-MYC to a similar extent. c-MYC protein is linked to drug resistance in leukaemia cells and found to be involved in the microenvironment-mediated drug resistance [30]. Such downregulation might be linked to the downregulation of the MAPK pathway, specifically through inhibition of MAPK14. Furthermore, the downregulation of CDK4 might be attributed to the downregulation of c-MYC [31]. Inhibition of CDK4 by JCBV is probably directed by synergizing the MAPK pathway regulation and c-MYC downregulation, but in the case of MEL, the downregulation of CDK is directed only by the downregulation of c-MYC [32].

Increased expression of JUN in leukemic cells treated with either MEL or JCBV was evident. JUN is involved in the expression of the FAS/FASL ligand, thus leading to apoptosis by the FAS receptor. Moreover, increased JUN expression provides positive feedback to enhance AP-1 activity [33]. The JNK pathway activates two major down-stream signaling pathways: death signaling such as c-JUN and FOS, and apoptosis signaling such as BAX. As an important JNK effector, AP1 induces cancer differentiation but several aspects of c-JUN activity support a role in the prevention of tumorigenesis and inducing apoptosis. The former suggests that c-JUN plays a role in apoptosis, and is stimulated by multiple extracellular matrix and genotoxic agents. Much of these triggers stimulate c-JUN N-terminal kinases, which give rise to the JUN protein phosphorylation and boost AP-1-dependent gene transcriptional activity [34]. c-JUN activates Cyclin D1 promoter and JUN B suppresses it. As reported previously cyclins together with CDK4/6 induce cells from G0 to S1 [35], however, in the current study, it was observed that CDK4 was inhibited, thus inhibiting cell division.

Such observation may suggest that MEL plays a key role in the cytotoxic impact of JCBV on cells. Nevertheless, MEL induces apoptotic cell death when administered solely, however, necrotic cell death is more evident when JCBV is administered. The transcriptional signature of MEL and JCBV are distinct, thus thorough profiling of the impact of BV and its constituents on cell death modes is needed.

## Conclusion

Honeybee venom is globally available and considered cost-effective and easily accessible. The tested Jordanian bee venom in comparison to MEL have demonstrated significant efficacy in inducing apoptosis, necrosis, and cell cycle arrest in myelogenous K562 human leukaemia cell line. The presented findings suggest that the tested honeybee venom and its constituents, particularly MEL, could be further developed for the treatment of CML leukaemia. The Jordanian bee venom was found active in inducing CML cell death via modulation of NF-κB and MAPK pathways, CDK4 inhibition, as well as upregulation of JUN and TNF genes. Nevertheless, the study suggests correlation between percentage of MEL in crude bee venom may be due to the time of collection or probability of high percentage of protease enzyme. The adoption of MEL as a potential alternative or supplementary CML therapy strategy needs further investigation. The key to understanding crude BV and MEL warrants further research to be clinically translated.

## Data Availability

All data generated or analyzed during this study are included in this published article.

## Abbreviations

AP-1: Activator protein 1 (JUN)
BV: Bee Venom
BAX: Bcl-2 Associated X-protein
BCR-ABL: Breakpoint cluster region-Abelson
BCL-2: B-cell lymphoma 2
CML: Chronic myelogenous leukemia
C-MYC: C-myelocytomatosis oncogene
CDK4: Cyclin-dependent kinase 4
DOX: Doxorubicin
JCBV: Jordanian crude bee venom
MAPK14: Mitogen-activated protein kinases
MEL: Melittin
NF-KB: Nuclear factor -kappa-B cell
STAT: Signal transducers and activators of transcription
TNF: Tumor Necrosis Factor
5-FU: 5-Fluorouracil
β-actin: Beta actin
